# Spatiotemporal Epidemiology of Female Thyroid Cancer: A Retrospective Population-Based Registry Study in the Hamadan Province, Iran (2010–2019)

**DOI:** 10.34172/aim.34951

**Published:** 2025-12-01

**Authors:** Erfan Ayubi, Shiva Borzouei, Sharareh Niksiar, Salman Khazaei

**Affiliations:** ^1^Social Determinants of Health Research Center, Institute of Health Sciences and Technology, Hamadan University of Medical Sciences, Hamadan, Iran; ^2^Cancer Research Center, Institute of Cancer, Hamadan University of Medical Sciences, Hamadan, Iran; ^3^Department of Endocrinology, School of Medicine, Hamadan University of Medical Sciences, Hamadan, Iran; ^4^Hamadan Cancer Registry, Hamadan University of Medical Sciences, Hamadan, Iran; ^5^Research Center for Health Sciences, Institute of Health Sciences and Technology, Hamadan University of Medical Sciences, Hamadan, Iran; ^6^Dental Implants Research Center, Avicenna Institute of Clinical Sciences, Hamadan University of Medical Sciences, Hamadan, Iran

**Keywords:** Hamadan, Iran, Spatiotemporal pattern, Thyroid cancer, Women

## Abstract

**Background:**

Thyroid cancer (TC) incidence varies regionally in Iran, with a notable increase observed in females. However, region-specific spatiotemporal epidemiological data are limited. This study aimed to quantify the spatiotemporal trends and geographic clustering of female TC in the Hamadan province, western Iran, from 2010 to 2019.

**Methods:**

Female TC cases from the Hamadan province were obtained from the population-based cancer registry. County-level standardized incidence ratios (SIRs) were calculated to adjust for differences in population size, and were smoothed using a hierarchical Bayesian spatial smoothing model that accounts for spatial and temporal dependence. Temporal trends were analyzed using joinpoint regression. Spatiotemporal clusters were identified using space-time scan statistics.

**Results:**

The incidence of female TC showed an average annual increase of 14.5% (Average annual percent change [AAPC]: 14.5, 95% confidence interval: 4.7 to 25.3; *P*<0.001) from 2010 to 2019 in the Hamadan province. The smoothed SIRs indicated increasing trends in northern and central counties, including Hamadan, Asadabad, Famenin, Razan, and Tuyserkan, while decreasing trends were observed in southern counties such as Nahavand and Malayer. A significant high-risk spatiotemporal cluster was identified in the Hamadan county during 2018–2019 (observed-to-expected cases ratio: 2.24, *P*<0.001), and a low-risk cluster was detected in Nahavand, Malayer, and Tuyserkan from 2010 to 2013 (0.51, *P*<0.001).

**Conclusion:**

This study revealed significant spatiotemporal heterogeneity in female TC incidence in the Hamadan province. Identification of high-risk clusters provides an evidence base for targeted preventive measures and health resource allocation.

## Introduction

 Thyroid cancer (TC) shows a pronounced gender disparity, ranking as the fifth most common cancer among women globally with an incidence rate three times higher than men.^1^ The highest incidence of TC occurs in developed countries.^1,2^ However, its incidence is increasing in underdeveloped and developing countries, such as in North Africa and the Middle East, where it has risen by 396% between 1990 and 2019.^3^ When stratified by country, the incidence of TC varies across North Africa and the Middle East, with Iran representing one of the countries with the highest incidence rates in women.^3^ The statistics show that in Iran, the age-specific incidence of TC has increased by 131% between 1990 and 2019.^4^

 There is a geographical inequality in the distribution of TC within Iran. The provinces in the west and central regions have the highest prevalence rates, while those in the east have the lowest.^5-7^ Although there exists provincial inequality in the occurrence of TC in Iran, the rates in finer geographic units such as county may have a different distribution than that at larger scales such as province, known as ecological fallacy.^5,8,9^ To accurately compare rates across different geographic units, the differences in population size should be adjusted through standardization.^10^ Additionally, from the perspective of spatial and spatiotemporal epidemiology, rates in neighboring geographic and temporal units often show similar values and tend to cluster due to spatial and temporal autocorrelation. Ignoring these autocorrelations when estimating rates for geographic units on a map can result in predicted variability that does not match observed data, a problem known as overdispersion.^11,12^

 One common method for identifying spatial, temporal, or spatiotemporal clusters of an outcome on a map is the use of Kulldorff’s spatial scan statistic.^13^ This statistic accurately detects locations where cases cluster across space and time while adjusting for issues such as overdispersion. Another effective approach for estimating rates in geographic units over time is the use of Bayesian hierarchical spatial models, which incorporate random spatial and temporal effects into conventional models, such as Poisson regression, to account for spatiotemporal autocorrelation.^14-16^ In Iran, studies on the geographic inequality of TC in women and the use of statistical and methodological methods such as space time scan statistics and Bayesian hierarchical spatial models to investigate this cancer at the county level remain limited. Identifying high risk areas and clusters is important from evidence-based policymaking perspective, as it allows health policy makers to efficiently allocate resources design targeted interventions in truly needy areas.

 Studying TC in the Hamadan province, located in western Iran, is important due to the convergence of potential risk factors, including a high level of obesity,^17^ a historical burden of iodine deficiency (as evidenced by a total goiter rate > 10%)^18^ and documented environmental exposure to elevated nitrate levels in soil, groundwater, and agricultural products.^19-21^ This unique combination of risk factors provides a strong rationale for investigating the spatiotemporal epidemiology of TC in this province. Considering the above issues, this study aimed to quantify the spatiotemporal trends and geographic clustering of female TC in the Hamadan province, western Iran, from 2010 to 2019.

## Materials and Methods

###  Design and Data 

 This study complies with the guidelines of the Strengthening the Reporting of Observational Studies in Epidemiology (STROBE) for reporting observational study findings.^22^ We conducted a retrospective population-based registry study of female TC data in nine counties of the Hamadan province during 2010 to 2019. This province comprises the northern counties of Razan, Kabudarahang, and Famenin; the central counties of Hamadan, Bahar, Asadabad, and Tuyserkan; and the southern counties of Malayer and Nahavand.

 The female TC cases were obtained upon request from the Cancer Office located in the Health Deputy at Hamadan University of Medical Sciences. Briefly, the Cancer Office manages a population-based cancer registry in which data are collected using both passive and active methods from all public and private diagnostic and therapeutic centers, and conducts duplicate checks. The International Classification of Diseases (ICD) was used for coding tumor features. The female population size of each county during the study period was obtained from the statistics office, located in the Health Deputy at Hamadan University of Medical Sciences and was projected annually based on census data.

 The analysis is divided into three steps: Joinpoint trend analysis, standardization of incidence rates, spatiotemporal cluster detection and smoothing standardized incidence rates.

###  Joinpoint Trend Analysis 

 We performed the temporal trend analysis of the incidence rate of female TC in the Hamadan province using Joinpoint regression analysis, implemented with the Joinpoint Regression Program (version 4.2.0.2). This method identifies points in time (“joinpoints”) where a statistically significant change in the trend occurs. For each segment defined by these joinpoints, the annual percent change (APC) was estimated to quantify the yearly rate of increase or decrease in incidence. Additionally, the average annual percent change (AAPC) was calculated to summarize the overall trend throughout the entire study period. The APC and AAPC estimates were reported with corresponding 95% confidence intervals (CIs), and statistical significance was set at *P* < 0.05.

###  Standardization of Incidence Rates 

 The female TC cases in each county follows a Poisson distribution, and the standardized incidence ratio (SIR) can be calculated as follows:

 SIRi = Oi/Ei

 where O_i_ and Ei are the observed and expected number of female TC in county *i*, respectively. The Ei is obtained by multiplying the population size of that county by the overall female TC incidence rate in the entire province for the same year. The pattern of spatial dependence in the estimated SIRs was evaluated for each year using Global Moran’s I statistic.

###  Spatiotemporal Cluster Detection

 Kulldorff’s space-time scan statistic was applied to identify the spatial clusters of female TC over time. The data utilized for the analysis included the geographic coordinates of the spatial units under study (counties), the annual count of female TC cases, and the corresponding population size for each county and year. This allowed the spatiotemporal Poisson model in SaTScan to properly account for variations in case counts and population at risk across both space and time. The space-time scan statistic uses cylindrical windows, with circular or elliptical bases and heights representing the time dimension, to detect clusters in which the number of female TC cases exceeds what would be expected by chance. This statistic assumes that the number of female TC cases within each spatiotemporal window follows a Poisson distribution and uses the log-likelihood ratio (LLR) to assess the significance of the detected clusters. The LLR function is defined as follows:


$$LLR=\frac{{\left(\frac{c}{E\left[c\right]}\right)}^{c}{\left(\frac{C-c}{E\left[C\right]-E\left[c\right]}\right)}^{C-c}}{{\left(\frac{C}{E\left[C\right]}\right)}^{C}}$$

Where *c* and *E[c]* correspond to the observed and expected number of cases within cylinder A, respectively. Likewise, *C* and *E[C] *represent the total observed and expected cases across the entire study area and time frame. The cylinder exhibiting the highest likelihood is designated as the primary cluster. The parameters applied for analysis were as follows: the probability model was chosen as discrete Poisson; the scanning window was a cylinder with a circular base; the analysis type was retrospective spatiotemporal; the maximum spatial and temporal cluster size was set to the default value or 50% of the population at risk and time, respectively; the number of Monte-Carlo replications was 999; and the significance threshold was established at a pseudo p-value below 0.05. The spatiotemporal cluster analyses were performed using the SaTScan software version 9.4.2.

###  Smoothing Standardized Incidence Rates 

 To smooth the SIR or relative risk (*θ_ij_*) of female TC across counties over time, the BYM and Random Walk of order 2 (RW2) models were used. The BYM model decomposes the *θ_ij_* into three components: A) baseline risk (α), B) Spatially structured component (uᵢ), assumes the risk in adjacent areas are related and accounts for the sharing information among neighboring areas. The uᵢ is modeled using a spatial conditional autoregressive (CAR) model. C) Spatially unstructured component (vᵢ), which represents random effects that are independent and identically distributed (i.i.d) across areas, with no spatial correlation.

 Temporal effects also include two components: Unstructured temporal effects (*φ_j_*), which is i.i.d. Structured temporal effects (*γ_j_*), which is modeled using the RW2 model. The RW2 model assumes the temporal changes follow a second-order random walk process and smooth temporal changes. After including the spatial and temporal random effect, the *θ_ij_* is modelled as follow;


$$\log\left(\theta_{ij}\right)=\alpha +v_{i}+u_{i}+\gamma_{j}+\emptyset_{j}$$

The aforementioned equation can be expanded to incorporate a term representing the interaction between spatial and temporal factors (*δ_ij_*). Here, we consider the interaction between structured spatial and temporal effects, and the model is defined as follows:


$$\log\left(\theta_{ij}\right)=\alpha +u_{i}+v_{i}+\gamma_{j}+\emptyset_{j}+\delta_{ij}$$

The prior distributions for the hyperparameters (σ) of the spatial and temporal random effects were specified using the default settings, which correspond to a log-gamma distribution with parameters (1, 0.00005). The adjacency matrix of the counties was based on the queen contiguity criterion. The posterior mean and uncertainty measures of the SIR were calculated using 95% credible intervals (CrI). The model convergence and adequacy were assessed using deviance information criterion (DIC), Watanabe-Akaike information criterion (WAIC), and the effective number of parameters. To assess the robustness of our spatial BYM model to the choice of priors, we conducted a sensitivity analysis by replacing the default priors with penalized complexity (PC) priors as proposed by Simpson et al.^23^ The hierarchical Bayesian spatial analysis is performed using the INLA package in R version 4.1.3.

 The study protocol was reviewed and approved by the by the research ethics committee of the Hamadan University of Medical Sciences. Data handling complied with applicable privacy and data protection regulations, and no identifiable patient information is reported in the manuscript.

## Results

**Figure 1:**
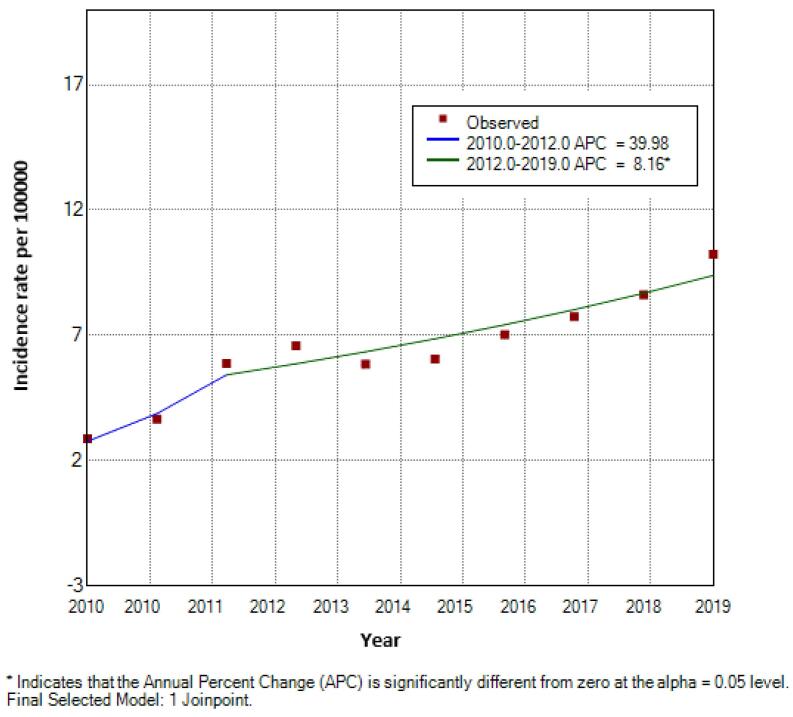
Trends in Crude Incidence Rate of Female Thyroid Cancer (2010–2019) Based on Joinpoint Regression Model

 Between 2010 and 2019, the crude incidence rate of female TC increased from 2.87 to 10.23 per 100,000. Joinpoint analysis identified one significant change point in 2012. From 2010 to 2012, the APC was 40.0% (95% CI: –15.6 to 132.1; *P* = 0.10). From 2012 to 2019, the APC was 8.2% (95% CI: 3.5 to 13.0; *P* < 0.001), indicating a statistically significant upward trend. The AAPC for the study period was 14.5% (95% CI: 4.7 to 25.3; *P* < 0.001) (Figure 1).

**Figure 2:**
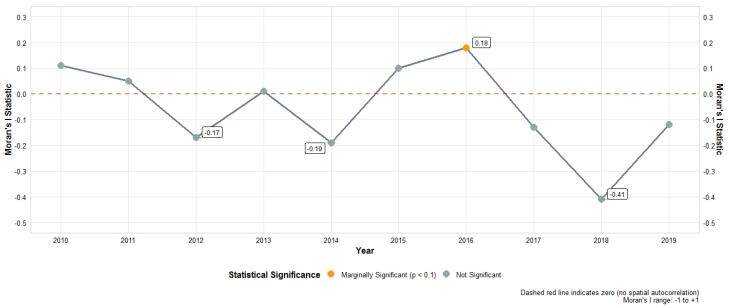
Spatial Autocorrelation of Female Thyroid Cancer Incidence (Standardized Incidence Ratio) in the Hamadan Province, Iran, 2010-2019 (Global Moran's I)

**Figure 3:**
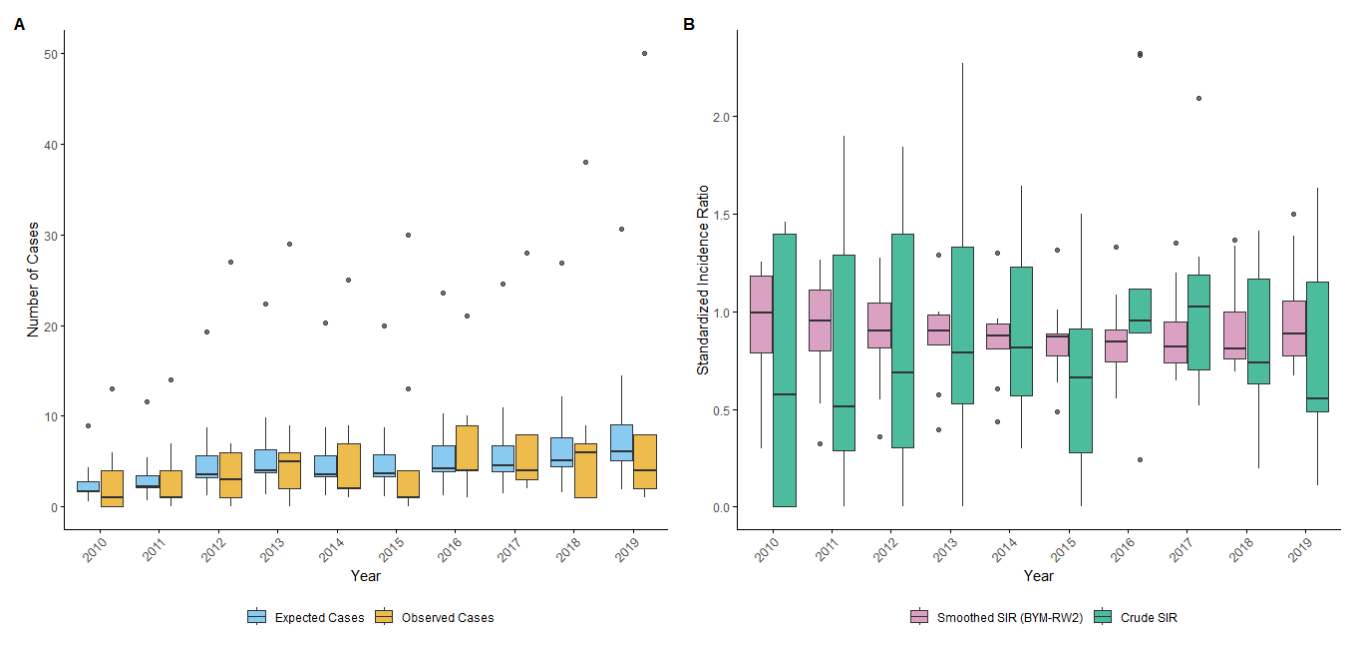
Distribution of Female Thyroid Cancer Metrics Across Counties in the Hamadan Province (2010-2019). (A) Boxplots of observed and expected cases. (B) Boxplots of crude and smoothed standardized incidence ratios (SIRs)

**Figure 4:**
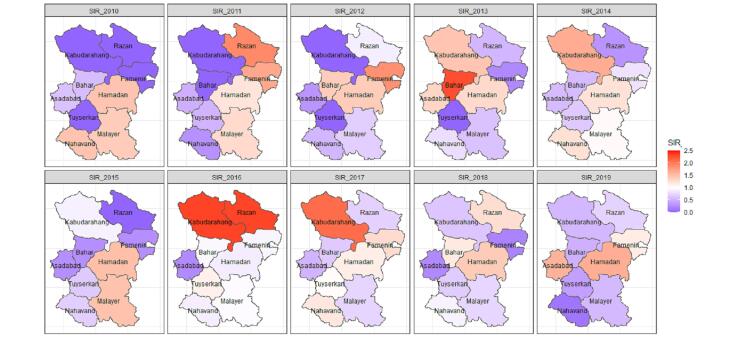
Crude Standardized Incidence Ratios (SIR) of Female Thyroid Cancer for Each County in the Hamadan Province During the Period 2010–2019

 The yearly Moran’s I values was a mix of positive and negative values, suggesting the presence of spatial autocorrelation and potential clustering in the crude SIRs. However, many of the p-values did not reach statistical significance (Figure 2). The observed cases, expected cases, crude SIR, and smoothed SIRs for the entire Hamadan province during 2010 and 2019 are presented in Figure 3. Between 2010 and 2019, the total number of observed cases was 557.00. Between 2010 and 2019, the number of cases increased by a factor of 3.36; 25.00 cases in 2010 (crude incidence rate: 2.87 per 100,000) and 84.00 cases in 2019 (crude incidence rate = 10.22 per 100,000. The distribution of female TC metrics across the Hamadan province showed considerable temporal and spatial variation over the study period (2010-2019). The mean number of observed cases exhibited a generally increasing trend, rising from 2.78 (SD = 4.38, median = 1.00) in 2010 to 9.33 (SD = 15.51, median = 4.00) in 2019, while expected cases (E) showed a parallel increase from 2.78 (SD = 2.52) to 9.33 (SD = 8.70). The crude SIRs demonstrated substantial fluctuations across the study period, with mean values reaching their peak in 2016 (mean = 1.18, SD = 0.69) followed by a declining trend in subsequent years to 1.04 in 2017, 0.80 in 2018, and 0.80 in 2019. The SIR range was notably wide (0.00-2.32) across different counties and years, indicating substantial geographical heterogeneity. The mean Bayesian smoothed SIRs estimated using the BYM-RW2 model ranged from 0.30 to 1.50, showing less extreme variation than the crude SIRs and providing more stable estimates of SIR across the spatial-temporal domain, with the maximum smoothed SIR (1.50) observed in 2019. The crude SIR of female TC for each county in the Hamadan province from 2010 to 2019 are shown in Figure 4. The crude SIR in the counties of the Hamadan province fluctuated remarkably during the study period. The overall trend indicates that in most years, the counties of Hamadan, Malayer, and Razan had observed values higher than expected. For example, in Hamadan, except for 2016 (crude SIR_2016_ = 0.88), the SIRs were above 1.00 in other years. Similarly, in Razan, one of the highest crude SIR values was observed in 2016 (observed cases = 9.00, expected cases = 3.88, SIR_2016_ = 2.31). In most years, the counties of Bahar, Kabudarahang, and Famenin had crude SIRs of 0.00 or less than 0.30.

 The Bayesian hierarchical model with spatial-temporal structured effects showed lower DIC (410.03) and WAIC (419.48) values compared to the simple Poisson model (DIC = 422.58, WAIC = 423.87). This indicates improved model fit by accounting for spatial and temporal dependencies. The higher effective number of parameters in the Bayesian model (18-23 vs. ~2) reflects a reasonable level of model complexity relative to the 90 data points (9 counties over 10 years), further supporting the stability and adequacy of the Bayesian hierarchical model. The PC priors provide a principled approach to controlling model complexity and shrinkage. We compared the original model with the modified model using PC priors. The results showed no meaningful differences in parameter estimates or model fit criteria. Specifically, the DIC was 410.03 for the original model and 410.04 for the PC prior model, while the WAIC was 419.48 and 419.50, respectively. This indicates that the model inference is robust to the prior specification and that the default priors used in the original analysis provide stable and reliable results

**Figure 5:**
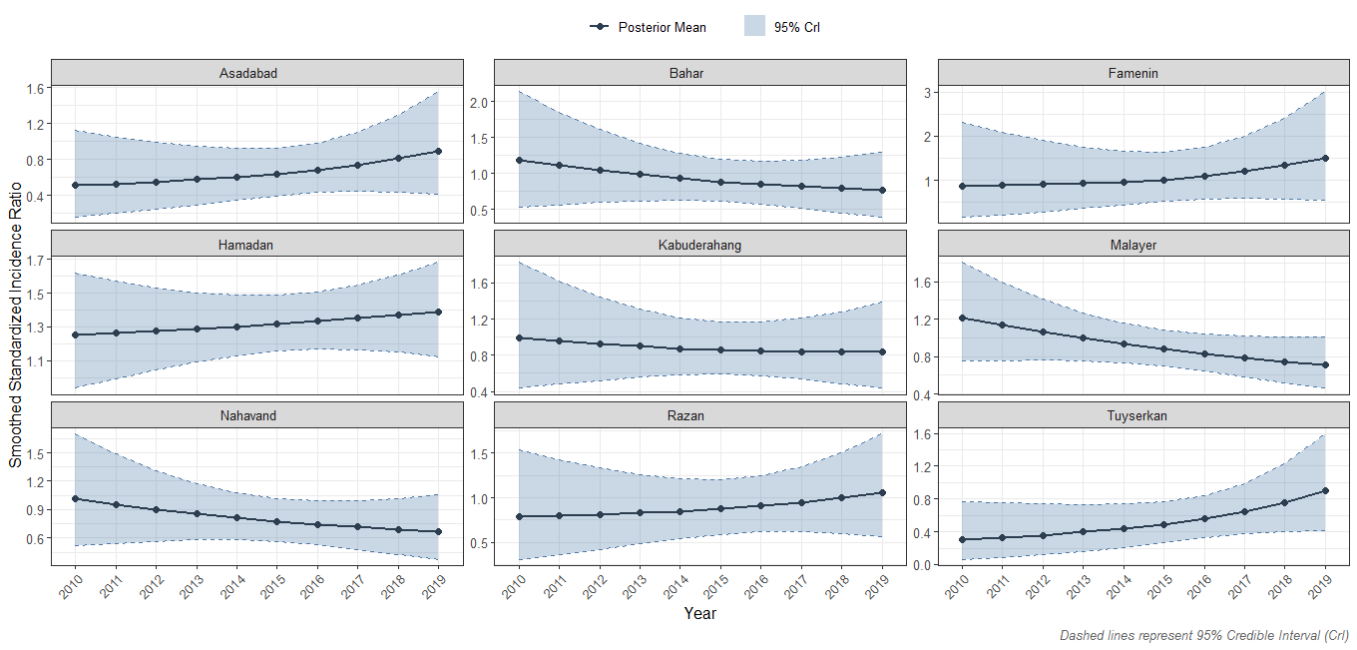
Smoothed Standardized Incidence Ratios (SIR) of Female Thyroid Cancer Across Counties in the Hamadan Province During 2010–2019, Posterior Mean with 95% Posterior Credible Intervals (Crl), Adjusted for Spatial and Temporal Random Effects

 The smoothed SIRs are presented in Figure 5. In the Asadabad county, it steadily increased from 0.51 in 2010 to 0. 89 in 2019. Similarly, in the Tuyserkan county the smoothed SIR increased from 0.30 in 2010 to 0.89 in 2019. The Razan county also showed an upward trend, with its smoothed SIR increased from 0.79 to 1.05 during the study period. Additionally, the Famenin county exhibited the highest growth, rising from 0.87 in 2010 to 1.50 in 2019. Hamadan county also showed a consistent increasing trend, with the smoothed SIR rising from 1.25 in 2010 to 1.38 in 2019. Conversely, some counties exhibited a decreasing trend in the smoothed SIR. For instance, the Bahar county’s SIR decreased from 1.18 in 2010 to 0.77 in 2019. The Kabudarahang county also experienced a gradual decrease, with its smoothed SIR dropping from 0.99 to 0.84. The Malayer county showed a downward trend, with its smoothed SIR falling from 1.21 in 2010 to 0.71 in 2019. Similarly, the Nahavand county demonstrated a decreasing trend, with the smoothed SIR declining from 1.01 to 0.67.

 Spatiotemporal analysis using Kulldorff’s space-time scan statistic revealed three significant clusters of female TC incidence. The primary high-risk cluster was in the Hamadan county (2018-2019), with a relative risk (RR) of 2.24 (*P* < 0.001). Two significant low-risk clusters were also identified: one in Malayer, Nahavand, and Tuyserkan counties (2010-2013) with an RR of 0.51 (*P* < 0.001), and another in Kabudarahang, Razan, Famenin, and Bahar counties (2010) with an RR of 0.08 (*P*= 0.007) (Table 1).

**Table 1:** Space-time Clusters of Female TC in the Hamadan Province During 2010-2019

**Cluster**	**County included**	**Time **	**Population size**	**O**	**E**	**Annual cases/100000**	**O/E**	**RR**	**logLR**	***P*** **-value**
Primary high rate cluster	Hamadan	2018-2019	323935	88	39.23	14.4	2.24	2.48	24.70	< 0.001
Primary low rate cluster	Malayer, Nahavand, Tuyserkan	2010-2013	291531	39	76.44	3.3	0.51	0.47	12.61	< 0.001
Secondary low rate cluster	Kabudarahang, Famenin, Razan, Bahar	2010	198534	1	13.08	0.5	0.08	0.07	9.64	0.007

O, observed; E, expected; RR, relative risk; logLR, log likelihood ratio.

## Discussion

 Between 2010 and 2019, the incidence of female TC in the Hamadan province increased by more than threefold. After incorporating the neighboring areas and temporal information, the SIRs trend shrunk toward null value. A high-risk cluster of female TC was identified in the Hamadan county, while low-risk clusters were detected in the southern counties including Malayer and Nahavand. An upward trend in incidence was observed in Asadabad, Famenin, Hamadan, Razan, and Tuyserkan counties, but declined in Malayer and Nahavand counties. In the Kabudarahang county, the SIR remained relatively stable.

 The trend analysis in the current study demonstrated that there was a 14.5% increase per year in the incidence rate of female TC. The rate of increase in Hamadan is particularly noteworthy when compared with local and national data. One study based on data from a national cancer registry program showed an APC of 6.95% for all, 10.03% for women, and -1.58% for men in Hamadan during 2014-2017.^7^ A study conducted in the Guilan Province, northern Iran, revealed that the incidence of female TC increased by approximately 24.6% annually (APC: 26.4; 95% CI: 22.5-30.4). Moreover, this upward trend was more pronounced among women compared to men.^24^ Another study conducted in the Golestan province in northern Iran showed that the incidence rate of TC increased at an average annual rate of 2.76% during the period from 2004 to 2013 (AAPC = 2.76; 95% CI: -3.68 to 9.64).^25^

 A national cancer report covering 2014-2017 demonstrated that the rate of female TC in Yazd, Isfahan, Fars and Kohgiluyeh and Boyer Ahmad provinces is higher than all other provinces. Nevertheless, it revealed a heterogeneous spatial distribution of TC incidence among women at the county level across most provinces of Iran. Specifically, for the Hamadan province, the spatial map from that report revealed intra-provincial variation,^5^ a pattern that our finer-scale analysis confirms and elaborates upon.

 The absence of directly similar studies on TC using the methodologies as applied in this study such as space time scan statistic and hierarchical Bayesian spatial smoothing model at the province level presents a fallacy for contextualizing the observed results. However, the successful application of such methodologies in the spatiotemporal analyzing of other cancers (e.g. colorectal and gastric cancer,^9^ breast cancer^8,26^ and childhood cancers^27^), which face similar challenges of small-area data problem, validates our analytical choice. Then, although the generalizability of our findings is limited by the provincial scale, the scalability of the applied methodologies might be considered in the future studies aiming to conduct spatiotemporal mapping of TC and other cancers at the provincial level.

 The yearly Moran’s I values include both positive and negative values, reflecting a dynamic spatial pattern that may vary over time due to complex epidemiological and environmental factors. While the corresponding *P* values generally did not reach statistical significance, it is crucial to distinguish between public health importance and statistical significance when interpreting the results. The positive or negative Moran’s I values may highlight meaningful spatial patterns relevant for public health interventions. Such patterns may not always be detected by classical tests due to limited statistical power but can have important real-world implications. These considerations highlight the value of the Bayesian spatiotemporal modeling framework employed in our study, which integrates spatial and temporal dependencies more comprehensively than Moran’s I test alone. This advanced approach improves our ability to explore the spatiotemporal patterns of female TC, providing a better understanding of the disease dynamics beyond what classical spatial statistics can offer.

 The shrinkage observed in the SIRs suggests that the changes in the trend of TC incidence rates may be influenced by spatiotemporal autocorrelation, random fluctuations, and spatial-temporal characteristics. Another application of using hierarchical Bayesian spatial method is their ability to address the small area estimation problem. Specifically, by incorporating information from the neighboring areas and temporal data, this method improve the precision of rate estimates, particularly in regions with limited data, such as counties with zero or only one registered female TC during a given year.^16^ Additionally, it is worth noting that even in contexts with an excess of zeros, Bayesian zero-inflated Poisson models remain effective for area cancer risk estimation and mapping.^28^ We used the BYM and RW2 models with an assumption of interaction between structured spatial and temporal effects. Additionally, a queen contiguity matrix and default value for hyperparameters were applied. However, for future TC mapping studies with larger datasets, it is recommended to explore different modeling scenarios. This includes applying different precision parameter priors, spatial random effects like Besag or BYM2, alternative temporal random effects such as RW1, and also using various types of adjacency matrices, for example those based on Euclidean distance.

 The incidence of female TC in the Hamadan province increased threefold between 2010 and 2019. Part of this increase may be attributed to improvements in the socioeconomic status (SES). Studies have provided evidence of a direct association between the Human Development Index (HDI) and the incidence of TC.^29^ The HDI in the Hamadan province was 0.736 in 2010 and 0.761 in 2019.^30^ Improvements in SES are expected to be associated with increases in health literacy and the utilization of diagnostic services.^31-33^ In the Hamadan province, the temporal trend of female TC varied across different counties. The temporal difference in TC incidence may result from temporal changes in factors such as overdiagnosis, the registration and notification system, demographic characteristics, clinicopathologic and pathogenic factors, as well as individuals’ lifestyles.^34-37^ It is worthwhile to decompose the difference in female TC incidence between 2010 and 2019 and estimate the contribution of each of the aforementioned factors to the observed temporal variation.^38^ The county of Hamadan is the capital of the Hamadan province (a referral city), where most healthcare services are concentrated and also the SES is higher compared to other counties, so it is expected that TC is more frequently diagnosed and reported in this county. Some studies have provided evidence regarding the association between environmental factors such as air pollution and radiation exposure and TC.^39,40^ In the city of Hamedan, the levels of such pollutions are also higher than threshold limit values.^41,42^

 Several limitations should be taken into account. The study results may be influenced by the ecological fallacy, the modifiable areal unit problem (MAUP), and edge effects. While these limitations are acknowledged, no formal sensitivity analysis for spatial aggregation (MAUP) or advanced methods for controlling edge effects (such as external adjacency) were performed. Therefore, the findings should be interpreted with caution considering these inherent spatial data challenges. The role of overdiagnosis in the possible exaggeration of rates in the Hamadan county should be taken into consideration. However, due to lack of data on screening rates or related proxy variables, we were unable to adjust for this potential confounder statistically. The spatiotemporal mapping was univariate; after examining the effect of confounding variables such as SES, the mapping may change. Due to the low number of cases per county and year, the sample size in this study is limited, which may lead to instability in rate estimates despite the use of advanced statistical methods such as hierarchical Bayesian modeling. No formal power calculation was performed for SaTScan. Since SaTScan is a nonparametric method based on probabilistic spatial-temporal models, classical power analysis methods are not directly applicable. Therefore, the results should be interpreted with caution given this limitation. Future studies with larger sample sizes or simulation-based power assessments are recommended to enhance cluster detection. Another limitation of our spatial cluster analysis is the relatively small number of spatial units (9 counties), which reduces but does not eliminate the risk of multiple testing issues inherent in cluster detection methods. Although we applied non-overlapping clustering in SaTScan and used robust Monte-Carlo *P* values, formal multiple testing corrections were not implemented. Future studies with larger spatial domains should consider such adjustments to further control false positive rates.

## Conclusion

 In conclusion, our analysis revealed varying spatiotemporal patterns of female TC across the Hamadan province between 2010 and 2019. Several high-rate and low-rate clusters, with some areas showing relative risks greater than 2 were identified. These patterns provide descriptive insight into the geographical distribution of female TC in the province and highlight areas where further investigation is warranted. Further research is needed to understand the underlying factors contributing to these observed patterns.
